# Rare case of exogenous *Candida dubliniensis* endophthalmitis: a case report and brief review of the literature

**DOI:** 10.1186/1869-5760-4-11

**Published:** 2014-05-02

**Authors:** Brian Douglas McMillan, Gary James Miller, John Nguyen

**Affiliations:** 1 Department of Ophthalmology, West Virginia University, 1 Stadium Dr, PO Box 9193, Morgantown, WV 26506-9193, USA

**Keywords:** *Candida dubliniensis*, Exogenous endophthalmitis, Enucleation, Ocular trauma

## Abstract

**Background:**

*Candida dubliniensis* is a recently described opportunistic fungal pathogen that rarely infects the eye. Reported cases of *C. dubliniensis* endophthalmitis have been of endogenous etiology and demonstrated recovery of visual acuity with timely treatment. We herein report an unusual case of severe *C. dubliniensis* endophthalmitis requiring enucleation.

**Findings:**

This is a retrospective, descriptive case report with a brief literature review. A 41-year-old Caucasian man, with a history of blunt trauma 8 months prior, presented to the emergency department with left eye pain and loss of vision 2 days after complicated cataract surgery. He was first evaluated by an outside ophthalmologist 3 months after trauma for left eye pain and progressive vision loss. He was found to have light perception vision with non-granulomatous anterior uveitis but no sign of ruptured globe. A dense cataract developed while he was treated with topical and subtenon's corticosteroids for which he underwent cataract surgery. Our examination revealed no light perception vision with a relative afferent pupillary defect, elevated intraocular pressure, moderate anterior chamber reaction, pupillary membrane, vitritis, and choroidal thickening on B-scan ultrasonography. Diagnostic vitrectomy revealed purulent vitreal debris, retinal detachment with severe retinal necrosis, and choroidal infiltrates. Operative fungal cultures grew *C. dubliniensis*. Despite intravitreal and systemic anti-fungal treatment, vision and pain did not improve, resulting in subsequent enucleation.

**Conclusion:**

*C. dubliniensis* endophthalmitis is uncommonly encountered and typically has reasonable visual outcomes. This is the first reported case of *C. dubliniensis*, likely exogenous endophthalmitis, resulting in enucleation, illustrating the potential virulence of this newly described organism.

## Findings

### Introduction

Fungal endophthalmitis can occur via either endogenous or exogenous route with *Candida* infections most commonly occurring from the endogenous route. *Candida* infections are predominately caused by *Candida albicans* species; however, several other species have been found to be pathologic especially in the immunocompromised state. *Candida* endogenous endophthalmitis often causes severe vision loss but rarely results in enucleation. In a study of 59 eyes with endogenous endophthalmitis, 56% of eyes with cultured positive yeast infection had a final visual acuity outcome of 20/200 or better, and only 3 progressed to enucleation, all of which were secondary to *Aspergillus*[1]. Exogenous fungal endophthalmitis is associated with poorer visual acuity outcomes and higher rates of enucleation given associated ocular trauma and direct inoculation of the eye; however, these cases are also most commonly caused by molds, fusarium, or fungal species other than *Candida*[2,3]. *Candida dubliniensis* is a relatively newly described species resulting in few reported cases of endogenous endophthalmitis, and most patients have reasonable visual acuity recovery [4-10]. We herein present an unusual case of severe *C. dubliniensis* endophthalmitis that required enucleation.

### Case report

A 41-year-old Caucasian man presented to the emergency department with left eye pain and loss of vision after complicated cataract surgery 2 days prior. His past ocular history was significant for blunt trauma to the left eye with a stick while riding an all-terrain vehicle 8 months ago; over the following 3 months, the eye became painful with deterioration of vision for which he sought ophthalmic care. At that initial examination, outside records indicated that his vision was light perception, and there was significant conjunctival injection, corneal edema, and anterior chamber reaction precluding a view of the fundus. There was no recorded sign of previous ocular trauma, keratic precipitates, hypopyon, or afferent pupillary defect, and his poor vision was attributed to the inflammation from initial injury. He was treated for non-granulomatous anterior uveitis with topical and subtenon's corticosteroid injection for several months. The lens developed progressive cataract changes with corticosteroid treatment, and he underwent cataract surgery that was complicated and unsuccessful due to significant sequela from uveitic changes. An intraocular lens was not placed due to questionable lens capsule integrity.

Our examination revealed a visual acuity of 20/30 right eye (OD) and no light perception of the left eye (OS). The right pupil was reactive to light, but the left pupil was fixed and non-reactive, with a left relative afferent pupillary defect. He had marked restriction of ocular motility of the left eye due to pain. The left upper eyelid was ptotic and edematous. The orbit was slightly tense to retropulsion, but no proptosis was appreciated. The intraocular pressures were 19 mmHg OD and 27 mmHg OS.

Slit-lamp biomicroscopy of the left eye revealed injected and chemotic conjunctiva. The cornea was hazy with diffuse fluorescein staining of the epithelium. The cataract wound incision was intact, and Siedel test was negative. The anterior chamber was without hypopyon, but moderate cells and flare were seen. A pupillary membrane precluded view to the fundus of the left eye. The patient's vital signs were normal. Complete blood count revealed an elevated white blood cell count (11,300 cells per dL) without a left shift.

**Figure 1:**
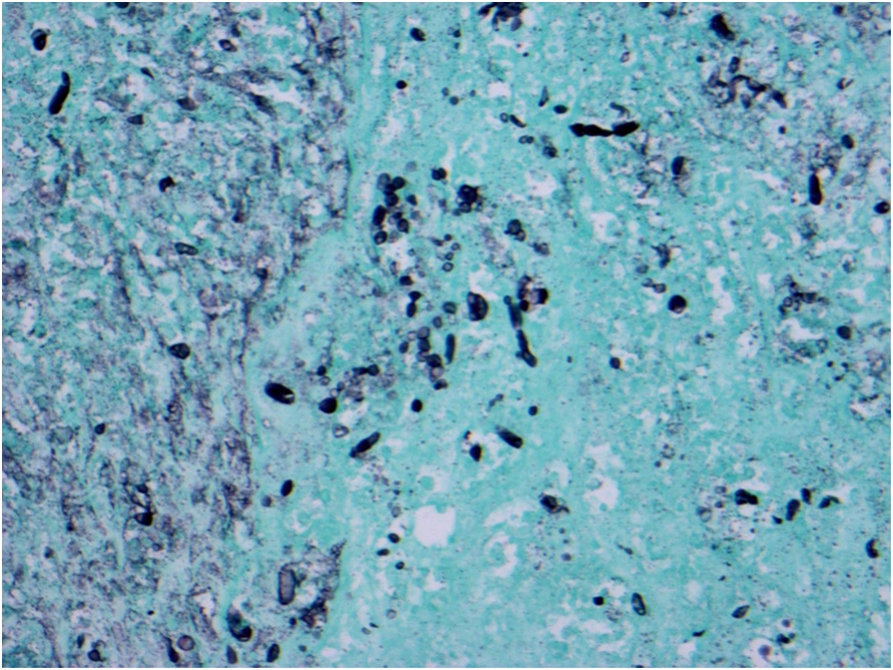
GMS stain illustrating hyphal elements.

B-scan ultrasonography of the left eye revealed diffuse vitritis and choroidal thickening. No retinal detachment was seen. The patient was admitted to the hospital for anterior chamber washout with removal of pupillary membrane, diagnostic vitrectomy, and intravitreal injection of vancomycin (1 mg/0.1 ml), ceftazidime (2.5 mg/0.1 ml), dexamethasone (0.4 mg/0.1 ml), and amphotericin B (0.4 mg/0.1 ml). Intraoperative findings included significant fibrin anterior chamber reaction, purulent material filling the vitreous cavity, funnel retinal detachment, and multiple choroidal infiltrates. Retinal detachment repair was not attempted due to the poor status of the retinal tissue. Initial gram stain revealed several polymorphonuclear cells with rare budding yeast and very rare pseudohyphae. Fungal culture subsequently revealed *C. dubliniensis* sensitive to amphotericin, caspofungin, and fluconazole (Figure 1). Serologic work-up for infection and inflammation included blood culture, rapid plasma reagin, fluorescent treponemal antibody-absorption, tuberculosis quantiferon gold, chest X-ray, angiotensin-converting enzyme, tick-borne disease panel, sedimentation rate, C-reactive protein, and human leukocyte antigen B27 which all were within normal limits.

The patient was treated with fluconazole 800 mg daily and evaluated by the infectious disease service who found no signs of extra-ocular infection. He had a normal cardiac exam, and additional cardiac imaging was not recommended by the infectious disease service. He adamantly denied intravenous drug use but was found to be hepatitis B and C positive without prior history of vaccination. Human immunodeficiency virus testing was negative, and hemoglobin A1c was 5.6. The patient did not recover his vision and continued to experience severe pain for which enucleation surgery was offered. He underwent enucleation surgery with placement of a porous implant without complication. He was treated for 4 weeks with fluconazole and had an uneventful recovery at 3-month follow-up.

### Discussion

Exogenous fungal endophthalmitis is usually caused by preceding fungal keratitis, trauma, and intraocular surgery, while endogenous fungal endophthalmitis is associated with recent hospitalization, systemic surgery, immunocompromised status, presence of intravenous lines, catheters, or intravenous drug use, organ transplant, and positive systemic cultures [11]. The mechanism of injury in this case is most likely due to a traumatic exogenous source in the setting of trauma. While he did not have an open globe injury, the late presentation to the outside provider and the chronic use of corticosteroid may have delayed the diagnosis of chronic fungal endophthalmitis. The patient's hepatitis B and C infection placed him at increased risk for an endogenous infection; however, he did not have needle tracks on physical exam and a history of recent intravenous drug use along with a positive blood culture to entirely suggest an endogenous cause. Another potential exogenous source of infection is from the cataract extraction 2 days prior to presentation. While both the initial trauma and cataract surgery could result in the infection, the typical latency period of weeks to months (averaged 1.8 months) after initial traumatic fungal inoculation may suggest that the initial trauma being more likely. However, exogenous fungal endophthalmitis, rare in the acute post-operative period, has been shown to develop as early as within a day of inoculation [2].

Over 77% of exogenous fungal endophthalmitis cases are caused by molds with yeast as the second most common (23%), specifically *C. albicans* as the most prevalent of the yeast species. There are, however, multiple species of *Candida* which can result in systemic infection in descending order of prevalence *C. albicans* (40%), *Candida glabrata* (26%), *Candida parapsilosis* (14%), *Candida tropicalis* (6%), *Candida krusei* (2%), *Candida lusitanie* (1%), and *C. dubliniensis* (1%) [12]. Those typically associated with endophthalmitis include *C. albicans* and *C. tropicalis*[1,11]. *C. dubliniensis* is a rare and newly described organism initially discovered in 1995 in a population of acquired immunodeficiency syndrome patients in Dublin, Ireland [4]. It has been reported to be pathogenic primarily in immunocompromised patients, HIV, IV drug use, and neutropenic patients, and most isolated strains are susceptible to azoles, amphotericin B, and echinocandins [13]. However, *in vitro* studies have indicated that resistance to fluconazole can develop with long-term exposure due to overexpression of multidrug transporter proteins in some *C. dubliniensis* isolates [8], but has not been reported in clinical settings.

*C. dubliniensis* is closely related phenotypically with *C. albicans* but can be differentiated by poor growth of *C. dubliniensis* at elevated temperatures (42°C and 45°C), chromographic culture techniques illustrated by CHROMagar *Candida* media (DRG International Inc., Springfield, NJ, USA) resulting in growth of dark green *C. dubliniensis* colonies and more specifically by species-specific polymerase chain reaction-enzyme immunoassay techniques [14]. These new technologies are helping to make the diagnosis of *C. dubliniensis* infections more readily with a subsequent increase in reported cases. The first case of endogenous *C. dubliniensis* endophthalmitis was presented in 2008 in a healthy 38-year-old male without immunosuppression or intravenous use history [5]. Pelegrin et al. subsequently described another case of endogenous C*. dubliniensis* endophthalmitis in a 41-year-old HIV-infected intravenous drug abuser in Spain in 2009 with good recovery of vision from 20/400 to 20/80. Espinosa-Heidmann et al. also reported a case of endogenous *C. dubliniensis* endophthalmitis in a 27-year-old man without immunosuppression with similar recovery of visual acuity after treatment. Moloney and Park described an additional five cases of *C. dubliniensis* endogenous endophthalmitis in patients with a history of IV drug use and hepatitis C. Four patients had final visual outcome of 20/60 or better, and one had hand motion vision. A last recent case published by Rosenberger et al. again illustrated excellent visual outcome of 20/25. While *C. dubliniensis* becomes an established, although rare, causative agent of endogenous fungal endophthalmitis, there has been no reported case of exogenous etiology nor a severe case resulting in enucleation upon our review.

In summary, we report the first case of *C. dubliniensis* endophthalmitis leading to eventual enucleation and likely the first case of exogenous etiology. While *C. dubliniensis* endogenous endophthalmitis cases have reasonable visual outcome, *C. dubliniensis* endophthalmitis can progress to cause severe intraocular destruction with complete loss of vision that requires enucleation, particularly in exogenous cases.

## Abbreviations

C. dubliniensis: *Candida dubliniensis*; OD: right eye; OS: left eye; NLP: no light perception.

## Competing interests

The authors declare that they have no competing interests.

## Authors’ contributions

BDM participated in obtaining clinical data, review of the literature, and drafting of the manuscript. GJM participated in the critical management in patient care and drafting of the manuscript. JN participated in the critical management in patient care and assisted in the literature review and drafting of the manuscript. All authors read and approved the final manuscript.
